# Non-Carrier Nanoparticles Adjuvant Modular Protein Vaccine in a Particle-Dependent Manner

**DOI:** 10.1371/journal.pone.0117203

**Published:** 2015-03-10

**Authors:** Arjun Seth, Fiona K. Ritchie, Nani Wibowo, Linda H. L. Lua, Anton P. J. Middelberg

**Affiliations:** 1 The University of Queensland, Australian Institute for Bioengineering and Nanotechnology, St. Lucia, QLD, Australia; 2 The University of Queensland, Protein Expression Facility, St Lucia, QLD, Australia; University of Massachusetts Medical Center, UNITED STATES

## Abstract

Nanoparticles are increasingly used to adjuvant vaccine formulations due to their biocompatibility, ease of manufacture and the opportunity to tailor their size, shape, and physicochemical properties. The efficacy of similarly-sized silica (Si-OH), poly (D,L-lactic-co-glycolic acid) (PLGA) and poly caprolactone (PCL) nanoparticles (nps) to adjuvant recombinant capsomere presenting antigenic M2e modular peptide from Influenza A virus (CapM2e) was investigated *in vivo*. Formulation of CapM2e with Si-OH or PLGA nps significantly boosted the immunogenicity of modular capsomeres, even though CapM2e was not actively attached to the nanoparticles prior to injection (i.e., formulation was by simple mixing). In contrast, PCL nps showed no significant adjuvant effect using this simple-mixing approach. The immune response induced by CapM2e alone or formulated with nps was antibody-biased with very high antigen-specific antibody titer and less than 20 cells per million splenocytes secreting interferon gamma. Modification of silica nanoparticle surface properties through amine functionalization and pegylation did not lead to significant changes in immune response. This study confirms that simple mixing-based formulation can lead to effective adjuvanting of antigenic protein, though with antibody titer dependent on nanoparticle physicochemical properties.

## Introduction

Vaccination has proved to be one of the most influential developments in human health history. Over years, vaccination has been based on live attenuated organisms, killed organisms or inactivated toxins. However, vaccines based on these traditional approaches suffer from problems including reversion to their virulent state or limited duration of protection [1,2]. These limitations have led to shifting of interest towards recombinant proteins such as subunit vaccines, based on a specific portion of the pathogen. Subunit vaccines are being preferred over attenuated live or inactivated whole organism vaccines as they are generally well purified and characterized, hence have improved safety profile and are easier to scale up over the latter. Despite the advantages of subunit vaccines, there are some downsides. For instance, usually antigen by itself is weakly immunogenic, which necessitates use of an adjuvant in formulation [2]. Selection of a suitable adjuvant is necessary to maintain balance between the upside enhancement of immunogenicity and the downside risk of side effects. In addition to enhancing immunogenicity, adjuvants can be employed to reduce the dosage or number of doses required for protective immunity.

In recent years, nanoparticles have attracted tremendous interest as a component within experimental vaccine formulations [3]. The use of nanoparticles in vaccinology is inspired by the fact that most pathogens have a dimension within the nano-size range [4], and therefore can be processed efficiently by the immune system, leading to a potent immune response. Nanoparticles are therefore being exploited to elicit desired immune responses for both prophylactic and therapeutic effects. They are utilized as either delivery systems to enhance antigen processing or to protect antigen from premature degradation, and/or as an immunostimulant to trigger immune response [5]. Nanotechnology allows customization of the properties of nanoparticles such as size, shape and surface charge to meet application requirements, resulting in a great variety of nanoparticles. A variety of biological as well as synthetic nanoparticles have been approved for human use [6–8], and many more are in clinical or pre-clinical studies [2,9]. Conventionally, the use of nanoparticles as a component in vaccine formulations is predicated on an assumed requirement for association between the antigen and nanoparticle components, to gain an adjuvanting effect [10,11]. This association between nanoparticles and antigen usually involves attachment either by conjugation, adsorption or encapsulation. However, a recent study has shown that it is possible to achieve an adjuvanting effect by simple mixing of nanoparticles and a sub-unit protein antigen from a virus-like particle, termed a capsomere, that has viral molecular architecture, which is being used in this study [12].

Virus-like particles (VLPs) are now a well-established vaccine class [6,13,14]. Modularized VLPs are emerging that allow design for efficacy against diseases different to the parent VLP through presentation of appropriate antigenic peptide modules within the VLP structure. VLPs are excellent vaccines as they are self-adjuvanting due to their particle characteristics and highly immunostimulatory because of their repetitive molecular structures [14,15,16,17]. Recent studies on the VLP-forming protein VP1 from murine polyomavirus (MuPyV) demonstrate that use of the sub-unit capsomere, the basic building block of a VLP, can lead to high immunogenic stimulation and protective efficacy with an otherwise immunologically weak peptide, M2e from influenza [18,19,12]. Studies using capsomeres based on papillomavirus [20], human respiratory syncytial virus [21] and human mucin-1 cancer antigen [22] also confirm that capsomeres are an emerging and interesting vaccine platform, which may retain the molecular activating signals of viruses but with some advantages including improved stability [23,24,25], tolerance to antigen incorporation [26] and simplified manufacture [18,27]. However, as capsomeres do not possess the particle structure of the VLP, formulation with adjuvant may be necessary, whereas good quality VLPs do not require adjuvant [18]. In this context, nanoparticles prove an interesting adjuvant class, as their addition to the vaccine formulation reintroduces the particle component that is inherent in VLPs but lacking in capsomeres, while the capsomere possesses appropriate and immunostimulatory molecular repetition.

Silica based nanoparticles are widely considered as being biocompatible, are non-toxic and possess flexible surface chemistry. These features make them attractive candidates for application in a vaccine system. Biodegradability is an important feature, which improves the safety profile of the formulation. PLGA and PCL are two of the most commonly used biodegradable and biocompatible polymers [2,28]. In particular, PLGA nps owing to their clinical approval and long safety record, have been widely explored and employed to develop nano-vaccines for a variety of antigens [29–32]. PCL is more hydrophobic and has a slower degradation profile as compared to PLGA [33].

A major goal of the work presented here was to understand, using *in vivo* tests, the impact of nanoparticle properties on their efficacy as non-carrier adjuvants for a modular capsomere sub-unit protein antigen. PEG-coated PLGA and PCL nps were synthesized by emulsion solvent evaporation. Highly monodisperse and spherical nps were prepared. Inorganic silica nps were obtained commercially. Viral modular capsomeres presenting M2e antigen were synthesized in a microbial expression system [19], and were formulated with the varied nps by simple mixing. The effects of varying the surface charge of silica nps on their adjuvanting activity, as well as the impact of selective PEGylation, were also investigated.

## Materials and Methods

### 1.1 Materials

Poly(D,L-lactic-*co*-glycolic acid) (PLGA, D,L-lactide:glycolide = 65:35, M.W. 40000–75000), polycaprolactone (PCL, M_n_ 70000–90000), (3-aminopropyl)triethoxysilane (APTES), o-phenylenediamine dihydrochloride, phosphate citrate buffer and HRP-conjugated goat anti-mouse IgG1 and IgG2a were purchased from Sigma-Aldrich (St Louis, MI) and used as supplied. 1,2-distearoyl-*sn*-glycero-3-phosphoethanolamine-*N*-[methoxy(polyethylene glycol)-2000](PEGPE) was purchased from Avanti (Alabaster, AL). Polyethylene glycol succinimidyl ester (mPEG-NSH; MW 5000) was purchased from Nanocs (New York, NY). Biotinylated M2e peptide was purchased from Peptide 2.0 Inc. (Chantilly, VA). Sodium chloride (NaCl), potassium chloride (KCl), disodium hydrogen phosphate (Na_2_HPO_4_), potassium dihydrogen phosphate (KH_2_PO_4_), Tween 20 and chloroform were obtained from Chem-supply (Gillman, SA, Australia) and used as supplied.

### 1.2 Protein expression and purification

Expression vectors for GST-tagged CapM2e (capsomere presenting M2e) and GST-tagged wt Cap (capsomere without M2e insert) were as described previously [18,19]. Expression vector was transformed into chemically competent *E*. *coli* Rosetta (DE3) pLysS cells (Novagen, Madison, WI). GST-tagged CapM2e and GST-tagged wt Cap were expressed and purified to give low-endotoxin (< 2 EU mL^-1^) CapM2e and wt Cap capsomeres, respectively, as previously described [19]. Endotoxin removal from GST-tagged CapM2e was performed by phase separation using Triton X-114 (X114, Sigma-Aldrich, USA) as previously described [19]. Endotoxin removal from wt Cap was performed by using an anion exchanger, a Vivapure Q Mini M spin column (Sartorius Stedim, France) as previously described [18]. Capsomere protein concentration was adjusted to 0.75 mg mL^-1^ with endotoxin-free PBS, and endotoxin content tested to be < 2 EU mL^-1^. Endotoxin level was analysed using LAL-based assay Endosafe PTS-2005 (Charles River Laboratories, Wilmington, MA). CapM2e and wt Cap capsomeres were aliquoted and stored in -80°C until further use.

### 1.3 Adjuvant Preparation

All adjuvant preparations were conducted in endotoxin free environment.

#### 1.3.1 Synthesis of PLGA and PCL nanoparticles

PLGA and PCL nps were prepared by an oil-in-water (o/w) emulsion solvent evaporation method as described previously [34]. Briefly, 400 μl of mixture of PLGA or PCL (4 mg) and PEGPE (8 mg) in chloroform was added dropwise into 4 ml water. Then the solution was sonicated at 10 W (Branson Sonifier 450 microtip probe ultrasonicator, Danbury, CT, USA) for four 25s bursts interspersed with cooling on an ice bath for 60s. The chloroform was separated from the emulsified solution by using a rotary evaporator (Rotavapor R-215, Büchi, Postfach, Switzerland). Nanoparticles were washed three times by centrifugation (18000 g, 5 mins) using Amicon ultra centrifugal filter devices (Millipore, Billerica, MA, USA) to remove free PEGPE. Endotoxin level in PLGA and PCL nps were analyzed using LAL-based assay Endosafe PTS-2005 (Charles River Laboratories, Wilmington, MA) and were found to be < 2.5 EU mL^-1^.

#### 1.3.2 Silica nanoparticle preparation

Commercial silica nps of nominal diameter 50 nm (Cat. 24040, Polysciences Inc., Warrington, PA) were dialyzed using snake-skin pleated dialysis membrane (nominal molecular weight cut-off of 10kDa; Thermo Scientific, Rockford, IL, USA) against PBS at 4°C for 24 h and adjusted to a nominal (based on the product label) silica concentration of 2 mg mL^-1^ with PBS. Endotoxin level in silica nps was analyzed using LAL-based assay Endosafe PTS-2005 (Charles River Laboratories, Wilmington, MA) and was found to be < 2 EU mL^-1^.

#### 1.3.3 Silica nanoparticles amine functionalization

Amine-functionalized silica nanoparticles (Si-NH_2_ nps) was prepared according to literature with some modification [35]. Commercial silica nps as above were dialyzed against Milli Q water at 4°C for 24 h and adjusted to a nominal silica concentration of 30 mg mL^-1^ with Milli Q water. Then, nanoparticle solution was subjected to centrifugal wash (18000 g, 20 mins) with absolute ethanol three times, followed by sonication (Branson Ultrasonics Corporation, Danbury, CT) at output 30 for 4 cycles of 20s to re-suspend in ethanol. Nanoparticle solution in absolute ethanol was incubated with 14% (v/v) (3-aminopropyl)triethoxysilane (APTES; Cat. A3648, Sigma-Aldrich, St Louis, MA) for 3h with constant stirring. After incubation, nanoparticle-APTES solution was subjected to centrifugal washing (18000 g, 20 mins) with absolute ethanol three times, followed by sonication to finally re-suspend in absolute ethanol. Then, amine functionalized nps were dialyzed against Milli Q water at 4°C for 24h and adjusted to a concentration of 2 mg mL^-1^ with Milli Q water. Endotoxin levels in amine-functionalized silica nps was analyzed using a LAL-based assay Endosafe PTS-2005 (Charles River Laboratories, Wilmington, MA) and was found to be < 2 EU mL^-1^.

#### 1.3.4 Silica nanoparticles PEGylation

Pegylated silica nanoparticles (Si-PEG nps) were prepared by adding polyethylene glycol succinimidyl ester (mPEG-NSH; MW 5000, PDI <1.08, purity >95%, Cat. PG1-SC-5k, Nanocs Inc) into 3.4 mg mL^-1^ Si-NH_2_ nps in 2:1 (mPEG-NSH:Si-NH_2_) molar ratio in an endotoxin-free environment. The mixture was stirred at room temperature for 2 h. Endotoxin level in pegylated silica nps was analyzed using LAL-based assay Endosafe PTS-2005 (Charles River Laboratories, Wilmington, MA) and was found to be < 2 EU mL^-1^.

### 1.4 Characterization of nanoparticles

#### 1.4.1 Dynamic Light Scattering and Zeta Potential measurement

The hydrodynamic diameter and surface charge (zeta potential) of nanoparticle solutions were measured with a Zetasizer Nano ZS (Malvern Instruments, Worcestershire, UK) using dynamic light scattering (DLS) and electrophoretic light scattering (ELS), respectively. This instrument employed a 633 nm laser wavelength at a scattering angle of 173° and 4 mW He-Ne laser power. For dynamic light scattering (DLS), nanoparticles were diluted 100-fold with water to avoid multiple scattering effects. Refractive index and viscosity of water were assumed to be 1.33 and 0.89 cP, respectively.

#### 1.4.2 Transmission electron microscopy (TEM) measurement

Two microliters of each sample (1 mg mL^-1^) was applied onto 200-mesh carbon-coated grids (Proscitech, Brisbane, QLD, Australia). Remaining liquid on the grids was blotted with filter paper after 2 min, and grids were washed with water, stained with 1% (w/v) uranyl acetate for 1 min and then allowed to air-dry prior to observation with a Jeol 1011 (Jeol Ltd., Tokyo, Japan) microscope at 100 kV accelerating voltage. Electron micrographs were recorded digitally using a side-mounted Morada camera (Olympus-Soft Imaging System GmbH, Münster, Germany) with iTEM software (version 3.2, Soft Imaging System GmbH).

### 1.5 Adsorption studies

50 μg of nanoparticle solution (Si-OH, Si-NH_2_, Si-PEG, PLGA or PCL nps) in PBS was mixed with 15 μg of CapM2e solution in PBS at room temperature. All tubes were centrifuged at 22000g, 4°C for 20 min. Supernatant was carefully removed and protein concentration was determined by high performance liquid chromatography (HPLC) analysis as described previously [36].

### 1.6 Immunization

Two biological studies were conducted. In the first study, six groups of five female BALB/c mice (Animal Resources Centre, WA, Australia) were immunized with 15 μg capsomere without M2e inserted (wt Cap) or CapM2e, alone or adjuvanted with 50 μg of Si-OH or PLGA or PCL nps. M2e peptide adjuvanted with Si-OH nps was used as control group. Adjuvanted CapM2e was prepared by mixing 15 μg CapM2e with 50 μg of selected adjuvant shortly prior to injection. Three subcutaneous immunizations were given on days 0, 21 and 42. Blood samples were taken by tail snip before the first immunization (day 0), followed by eye bleeds on days 14 and 35. Final blood sample was collected on day 56 by heart puncture. All animal experimental work was reviewed and approved by The University of Queensland Animal Ethics Committee (AEC Approval Number: AIBN/058/13/NIRAP/SMART FUTURES). All animals were cared for humanely in accordance with the Australian Code of Practice for the Care and Use of Animals for Scientific Purposes.

In the second study, four groups of five female BALB/c mice (Animal Resources Centre, WA, Australia) were immunized with 3 μg M2e peptide adjuvanted with 50 μg of Si-OH nps or 15 μg CapM2e adjuvanted with 50 μg of Si-OH or Si-NH_2_ or Si-PEG nps. The immunization regime was same as that in the first study. All animal experimental work was reviewed and approved by The University of Queensland Animal Ethics Committee (AEC Approval Number: AIBN/189/12/NIRAP/SMART FUTURES). All animals were cared for humanely in accordance with the Australian Code of Practice for the Care and Use of Animals for Scientific Purposes.

### 1.7 ELISA (Enzyme-linked immunosorbent assay)

Pierce Streptavidin High Binding Capacity (Thermo Fisher Scientific, Waltham, MA) coated plates were washed 3 times with PBST (137 mM NaCl, 2.7 mM KCl, 10.15 mM Na_2_HPO_4_, 1.76 mM KH_2_PO_4_, pH 7.4, 0.05% (v/v) Tween 20). Biotinylated M2e peptide (Peptide 2.0 Inc., Chantilly, VA) at 10 mg mL^-1^ in PBS was adsorbed to the plates, 100 μL per well for 2 h at room temperature. Plates were then washed 3 times with PBST. Plates were incubated with mouse sera initially at 100-fold dilution followed by four-fold serial dilutions with PBST containing 0.5% (w/v) skim milk (90 min, 37°C). After washing 4 times with PBST, HRP-conjugated goat anti-mouse IgG1 or IgG2a (Ab97240 or Ab97245, respectively, Abcam, Cambridge, UK) was added at 20 000- or 10 000-fold dilution, respectively, followed by incubation (90 min, 37°C). Plates were washed 4 times with PBST and developed (0.4 mg mL^-1^ o-phenylenediamine dihydrochloride (Sigma-Aldrich, St Louis, MA), 50 mM phosphate citrate buffer containing 0.03% (w/v) sodium perborate) prior to absorbance measurement at 450 nm. End point titers were determined as the highest dilution of serum for which the OD was 3 standard deviations above the mean optical density of blank wells.

### 1.8 ELISPOT (Enzyme-linked immunospot)

IFN-γ ELISPOT was performed as described previously [37]. Briefly, 14 days after final immunization, splenocytes were restimulated *in vitro* in the presence or absence of M2e peptide. The number of spots of cells secreting IFN-γ was counted to assess the frequency of M2e-specific cytotoxic T-cells. Concanavalin A (Con A) was used as non-specific positive control.

### 1.9 Statistical Analysis

Statistical analysis was performed using GraphPad Prism Version 5.03 (GraphPad Software Inc., USA). Comparison of more than two groups was performed with the Tukey’s multiple comparison tests. Comparison between two groups was performed with *t* test. *p* < 0.05 was considered statistically significant.

## Results and Discussion

The adjuvanting efficacy of similarly-sized nanoparticles having different properties was investigated in *in vivo* immunogenicity studies, particularly in their roles to adjuvant modular capsomeres. Modular capsomere presenting antigenic M2e peptide from Influenza A virus (CapM2e) was synthesized as described previously [18,19,12]. Formulations of CapM2e with nanoparticles were made by simple mixing prior to injection.

In the first study, adjuvanting efficacy of nanoparticles made of three different materials, biocompatible inorganic silica (Si-OH), biodegradable poly (lactic-co-glycolic acid) (PLGA) and polycaprolactone (PCL), were studied. Biocompatible inorganic solid silica nanoparticles were obtained commercially, whereas PLGA and PCL nanoparticles were prepared using an oil-in-water (o/w) emulsion solvent evaporation method by employing poly(ethylene glycol)-distearyl phosphoethanolamine (PEGPE) as the oily emulsifier during the process, as described [34]. The size of PEG-coated biodegradable nanoparticles could be controlled by varying the ratio of polymer to the oily emulsifier and also by varying sonication conditions.

**Fig 1:**
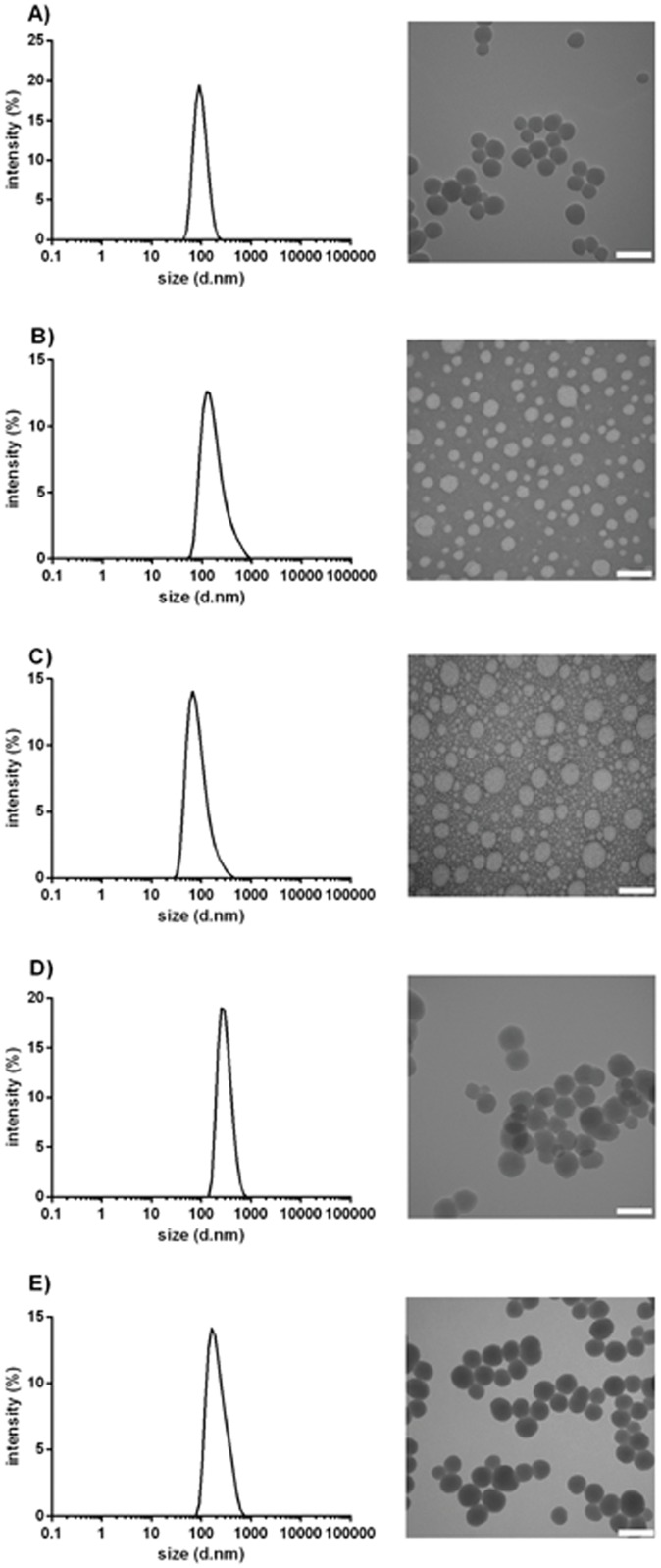
TEM images of nanoparticles A) silica, B) PLGA, C) PCL, D) amine functionalized silica and E) pegylated silica. Scale bar is 100 nm.

Fig. 1 shows the transmission electron micrographs (TEM) of Si-OH, PLGA, and PCL nanoparticles, revealing the spherical morphology of each type of nanoparticle. The average size of nanoparticles as observed from TEM is presented in Table 1. TEM images show that PLGA and PCL nanoparticles were more polydispersed as compared to Si-OH nanoparticles. Nanoparticle size was also determined using dynamic light scattering (DLS) analysis (Table 1), which gave a slightly larger size in comparison to TEM measurement. TEM gives information about size and surface morphology (i.e., shape and surface structure of individual nanoparticles) in a dried state while DLS measures the hydrodynamic diameter of particles in solution. This hydrodynamic diameter is larger than the actual dried diameter as it also takes into account any associated hydration layer as well as adsorbed organic stabilizers if present. Interpretation is also dependent on solution parameters including viscosity used to estimate the Stokes diameter in DLS.

**Table 1:** Characteristics of different nanoparticles.

Nanoparticles	Size (d_h_. nm)±S.D. (DLS)	Polydispersity Index (PDI)	Size (d_t_.nm) (TEM)
Silica nanoparticles (Si-OH)	81±2.3 nm	0.076	50 nm
PLGA nanoparticles (Poly(D,L-lactide-co-glycolide)	88±1.8 nm	0.130	40–60 nm
PCL nanoparticles (Poly caprolactone)	87±1.5 nm	0.240	40–70 nm

S.D.- Standard deviation, d_h_-hydrodynamic diameter

**Fig 2:**
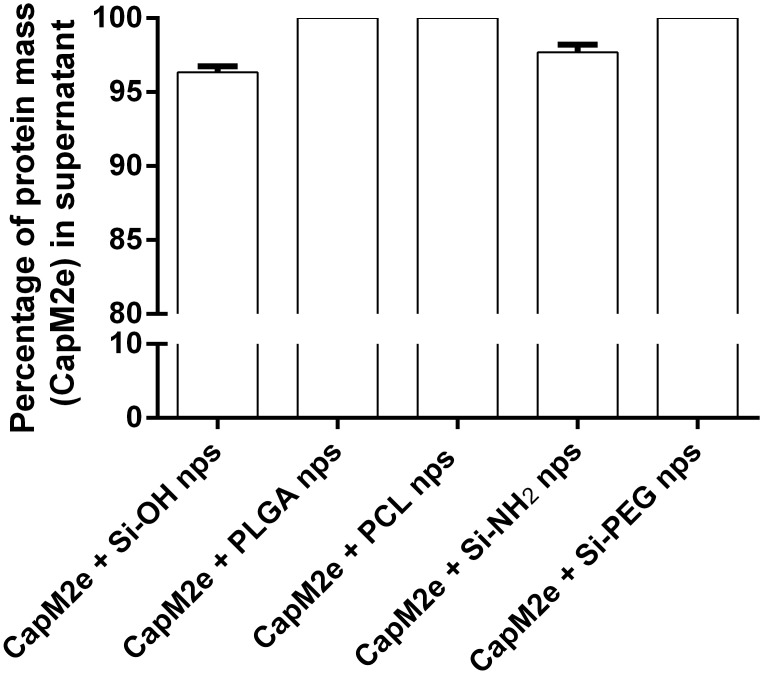
Percentage of protein mass (CapM2e) in supernatant after mixing with different nanoparticle solutions.

Previous studies have shown that 50 nm non-carrier nps can strongly adjuvant the viral sub-unit capsomere antigen, even in the absence of significant association between nanoparticles and antigen [12]. In the present study, since both CapM2e (isoelectric point *pI = 4*.*9*) [38] and Si-OH nanoparticle (Zeta potential = -41.3 mV) carry net negative charge at physiological pH, significant interaction between them was not expected and indeed was not found in previous studies [12]. Significant association between PLGA and PCL nps with CapM2e was also not expected as each np type includes polyethylene glycol (PEG) chains that will locate to the np surface and provide for low protein-binding character [39,40]. This expectation was confirmed by determining the extent of adsorption of these CapM2e onto these three sets of nanoparticles (Fig. 2).

**Fig 3:**
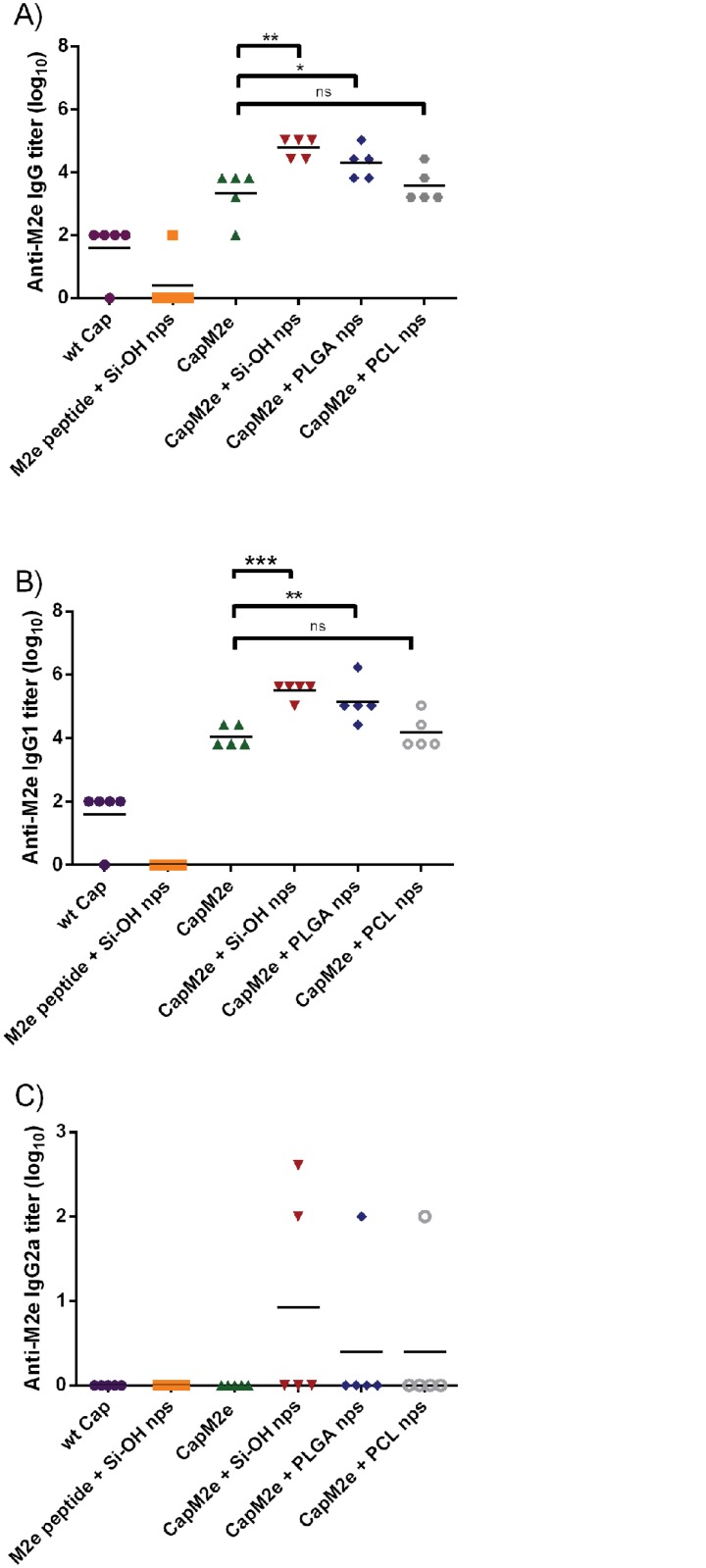
M2e-specific antibody titers in BALB/c mice following three subcutaneous immunizations with different formulations. A) Total IgG, B) IgG1, and C) IgG2a. *, p<0.05; **, p<0.01; ***, p<0.001; ns = not significant.

Fig. 3 shows the endpoint titer of anti-M2e specific total IgG, IgG1, and IgG2a in BALB/c mice after three subcutaneous immunizations with different formulations. Capsomere without M2e inserted (wt Cap) was injected as a negative control and, as expected, induced very low M2e-specific antibodies. A similar result was observed for the negative-control formulation of M2e peptide with Si-OH nps which also did not induce a specific anti-M2e immune response, confirming the low immunogenicity of this peptide sub-unit antigen. CapM2e without adjuvant induced a 10^4^ antibody titer, consistent with results observed earlier [19], revealing a significant effect of modularizing the weakly immunogenic peptide M2e into the molecular viral architecture provided by the capsomere, even in the absence of a particle adjuvant. Fig. 3A shows that the immunogenicity of CapM2e was boosted significantly, more than ten-fold, when formulated with Si-OH or PLGA nps. This adjuvanting effect however was not observed when CapM2e was formulated with PCL nps. PCL has a higher hydrophobicity and slower degradation profile in comparison to PLGA [41]. A number of studies have demonstrated that the hydrophobicity of nanoparticles could affect the overall immunogenicity of antigens [42–44]. Hence, the hydrophobic properties of PCL might affect the interaction of PCL with the cells of the immune system or with the modular capsomere which could then affect the mechanisms of antigen processing. It is interesting to observe that two biodegradable nanoparticles with different biodegradation profiles have very different effects on boosting (or not) the immune response of antigen.

**Fig 4:**
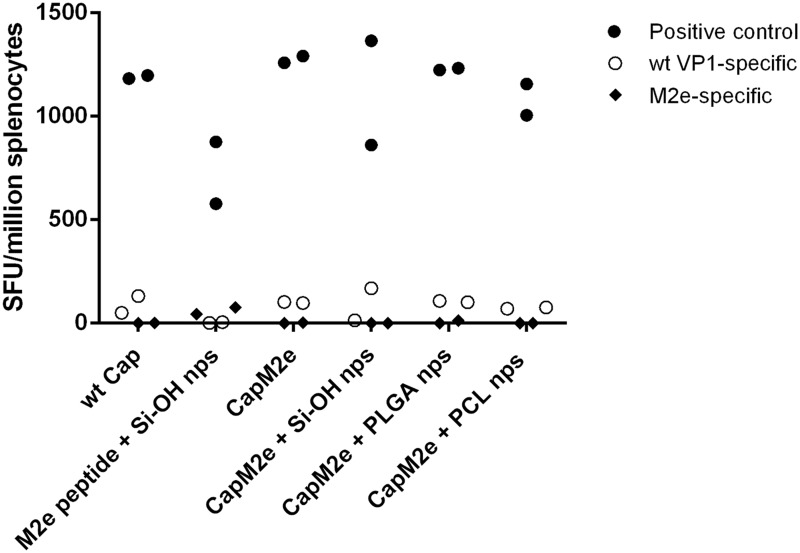
Determination of IFNγ response in splenocytes. M2e-specific T-cell response was evaluated by measuring IFNγ secreting cells by enzyme-linked immunospot (ELISPOT). A) Carrier (wt VP1)-specific, B) M2e-specific, C) positive control (Concanavalin A (Con A)). No significant difference was observed between the mean value of each group (p>0.05).

Fig. 3B and C show the anti-M2e specific IgG1 and IgG2a titers of immunized mice that correlate to Th2 (humoral-biased) and Th1 (cellular-biased) responses, respectively. The high IgG1 and low IgG2a titers reveal a Th2- biased immune response to capsomeres with as well as without nanoparticles. This observation is unsurprising as BALB/c mice are known to develop antibody-predominant immune responses [45]. Further study using Th1-skewed mouse strains, such as C57BL/6, would be needed to confirm whether Th2-biased immune responses observed here was due to the mouse strain used in this study, the antigen, or the formulation of CapM2e–nps. Other published studies related to M2e also resulted in an antibody-predominant immune response [46,47], while only a few studies have reported cellular response [48,49]. This observation was further confirmed by ELISPOT assay results (Fig. 4) which show a minimum number of cells secreting interferon gamma (IFN-γ).

Formulation of CapM2e with Si-OH nps induced the highest antibody titer in comparison to other formulations. Therefore, further study was conducted on Si-OH np variants, to reveal the effects of different surface properties on adjuvanting efficacy. Silica nanoparticles, inherently, have a negatively-charged surface due to the presence of silanol groups. To introduce positive charges on the surface of Si-OH, (3aminopropyl)triethoxysilane (APTES) was coated on the surface of Si-OH through formation of siloxane bonds by reacting with silanol hydroxyl groups. Si-NH_2_ was further modified to shield the surface charge by conjugation of polyethylene glycol (PEG). This charge transition on the surface of nanoparticles was assessed with zeta potential measurement, as presented in Table 2. The unmodified Si-OH nps showed an average zeta potential of-41.3 mV, which shifted to +44.5 mV after modification with APTES (Si-NH_2_). The zeta potential of Si-NH_2_ nps was reduced after conjugation with PEG derivative (+3.41 mV) which indicates overall surface coverage of nanoparticles by PEG chains. There was an increase in the size of nps after each modification step (Table 2).

**Table 2:** Characteristics of silica nanoparticles with different surface properties.

Nanoparticles	Size (d_h_.nm)±S.D. (DLS)	Polydispersity Index (PDI)	Zeta Potential (mV)
Silica nanoparticles (Si-OH)	81±2.3 nm	0.076	-41±2.1 mV
Silica nanoparticles with amine functionalization (Si-NH_2_)	103±2.7 nm	0.049	+45±1.4 mV
PEG-coated silica nanoparticles with amine functionalization (Si-PEG)	129±1.9 nm	0.130	+3.4±0.5 mV

S.D.- Standard deviation, d_h_-hydrodynamic diameter

**Fig 5:**
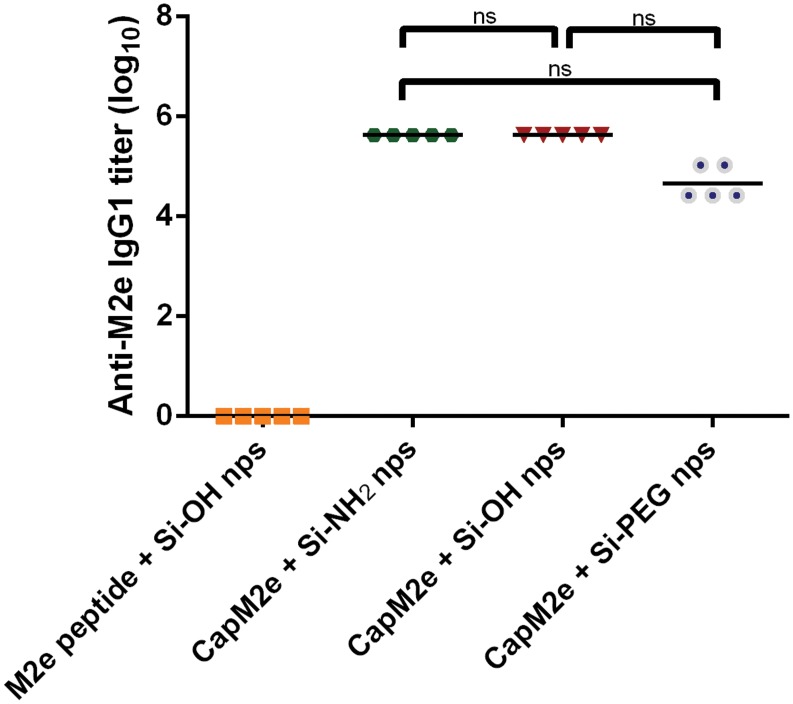
M2e-specific IgG1 titer in BALB/c mice following three subcutaneous immunizations with different formulations. *, p<0.05; **, p<0.01; ***, p<0.001; ns = not significant.

Fig. 5 shows the endpoint titer of anti-M2e specific IgG1 in mice after three subcutaneous immunizations with different formulations. No significant differences in immune response between different nanoparticle formulations with negative, positive and neutral surface charges could be observed statistically as these different groups were compared by one-way ANOVA followed by Tukey’s multiple comparison. Studies have reported that nanoparticle physicochemical properties including size [10,50], shape [51,52], surface charge [53,54] and hydrophobicity [43] influence the interaction of nanoparticles with the immune system [42]. Several *in vivo* studies have reported that surface charge of nanoparticles affects the immune response of formulations [55,56]. However, Foged and co-workers reported no effect of surface charge of nanoparticles of similar sizes on their uptake by dendritic cells (DCs) [53]. In this present study, the immune response might already be at its maximum and thus no further boosting of immunogenicity of formulation could be observed on changing the surface charge of nanoparticles. Further study is needed to fully explore and mechanistically understand these effects, for different antigen classes, and represents ongoing work in our laboratory.

This study explored the potential of various nanoparticles as a non-carrier adjuvant to modular capsomere, in contrast to a conventional attachment approach which involves loading of antigen either on the particle surface by chemical conjugation or physical adsorption or within the particle by encapsulation or entrapment. This non-attachment approach is increasingly being investigated [12,57], particularly as it offers much simpler formulation by mixing. Wibowo et.al [12] showed that non-carrier silica nanoparticles significantly boosted the immunogenicity of a subunit antigen even in the absence of significant association between these two components. However, a study by Zhang et al. [57] did not show enhanced IgG response by simple mixing. This difference is perhaps due to the absence of PAMPs in the OVA antigen that they used in comparison to viral capsomere used in the study by Wibowo et al. [12]. The presence of PAMPs in viral capsomere can act as danger signals that activate pattern recognition receptors on cells of the immune system [58]. Also, the difference in immunogenicity might be due to particle size (500 nm in Zhang et al. study, 50 nm in Wibowo et al.). Wibowo et al. clearly showed the striking effect of silica particle size on the adjuvanting efficacy, where boosting of immune response was observed for 50 nm sized silica nanoparticles but not for the larger (1000 nm) counterpart. Other studies have shown that size of particles is critical for their adjuvanting activity [10,59,60]. Nevertheless, Zhang et al. demonstrated that the addition of non-attached formulation to a nanoparticle-encapsulated antigen formulation enhanced antigen-specific IgG level and avidity, as well as increased cytokine secretion and memory T cell generation [57], highlighting the potential of nanoparticles as a non-carrier adjuvant class.

In this study, the various nanoparticles used have similar size but different physicochemical characteristics. The results demonstrated the superiority of silica nanoparticle in comparison to PLGA and PCL, possibly due to the ability of silica nanoparticles to induce several immunostimulatory cytokines, such as IFN0γ, IL-4 and IL-3 [61], or possibly because PLGA and PCL nanoparticles were not at their optimal size. Yan et al. [60] reported size-dependent adjuvanting effects for different particle types, and found that there exists an optimum size range for maximum adjuvanting efficacy for each type of particle.

This study further demonstrates the potential of the modular capsomere as a vaccine platform, as shown in previous studies [12,18,19]. A significant effect of modularizing the weakly immunogenic M2e antigen into the molecular viral architecture of a capsomere on immunogenicity was observed, suggesting that even in the absence of particle stimulation, capsomeres still retain the molecular activating signals of viruses. Moreover, nanoparticles can further enhance capsomere immunogenicity by introducing the particle component which is lacking in the capsomere structure. The low amount of endotoxin presence in the formulation suggests that the immunogenicity observed was not due to the bacterial signals. This two-component formulation prepared by simple mixing can potentially activate the immune system through two different pathways, leading to the observed strong immune response. This simple mixing approach, by eliminating the complexity of antigen conjugation or incorporation, may simplify approaches for the preparation of safe and efficacious nanoparticle-containing vaccines, though in a fashion that is nanoparticle and probably antigen specific.

## Conclusion

This study investigated the effects of composition and surface properties of non-carrier nanoparticles on their adjuvanting efficacy for modular capsomeres presenting influenza M2e antigen (CapM2e). Our findings showed that Si-OH and PLGA nps could boost the immunogenicity of these capsomeres, with the immune response being predominantly antibody biased and close to the maximum expected. It was observed that there was no significant difference on adjuvanting effect of Si-OH nps after varying their surface properties. Induction of maximal immune response by these nanoparticle adjuvanted modular capsomere formulations is attributed to combined synergistic effects of viral molecular and particle signals contributed by the capsomere protein and nanoparticle components, respectively. This simple mixed formulation of subunit antigen (capsomere) augmented with nanoparticles can prove to be a more robust formulation than for simpler antigens where in the absence of viral molecular signal, the properties of the adjuvant particle may become more critical. Understanding the mechanism of action of these formulations through further studies will be important for accelerating the rational design of nanoparticle-containing vaccines.
